# ﻿Genome sequencing provides novel insights into diadromous migration adaptations in the roughskin sculpin, *Trachidermus fasciatus* (Scorpaeniformes, Cottidae)

**DOI:** 10.3897/zookeys.1256.153772

**Published:** 2025-10-23

**Authors:** Tianwei Liu, Kun Huang, An Xu, Zhenming Lü, Li Gong, Jing Liu, Ming Tang, Liqin Liu

**Affiliations:** 1 National Engineering Laboratory of Marine Germplasm Resources Exploration and Utilization, College of Marine Sciences and Technology, Zhejiang Ocean University, Zhoushan 316022, China Zhejiang Ocean University Zhoushan China; 2 School of Life Sciences, Jiangsu University, Zhen jiang 212013, China Jiangsu University Zhen jiang China

**Keywords:** Comparative genomics, evolutionary adaptation, genome assembly

## Abstract

The ecological opportunities presented by the invasion from marine to freshwater habitats are considered to be an important catalyst for the adaptive radiation of many fish taxa. The sculpins (family Cottidae) serve as a typical representative of such successful evolutionary radiation from marine to freshwater environments, and among which, several intermediate diadromous taxa that can adapt to both marine and freshwater habitats have been evolved. However, little is known about the genetic basis underlying the diadromous migration adaptations for these euryhaline sculpins. Here, we have constructed a high-quality chromosome-level genome of the Cottidae species roughskin sculpin (*Trachidermus fasciatus*) and made a comparative genomic analysis of the genomes from the closely related species, aiming to elucidate the genetic basis and the possible evolutionary origin of its unique diadromous lifestyle. Our Hi-C data anchored the preliminary assembled sequences to 20 pseudochromosomes, resulting in a final corrected genome size of 526.39 Mb, with a scaffold N50 of 24.94 Mb. Phylogenetic analysis supports that the Cottidae family, to which roughskin sculpin belongs, possibly originated from the ocean, and should be reclassified under the suborder Cottoidei of the order Perciformes, rather than the classical order Scorpaeniformes. Comparative genomic analysis showed that 184 and 856 putative gene families in roughskin sculpin have undergone expansion and contraction, respectively. Enrichment categories of the top candidate genes under significant expansion were mainly associated with oxygen transport and immune response, possibly indicating their adaptations to the great oxygen demand and a wider range of pathogens across different salinity gradients during diadromous migration of roughskin sculpin. Additionally, 477 putative genes were identified to have undergone positive selection, which were enriched in categories related to DNA repair, innate immunity, and ion transport. This may indicate that the adaptive remodeling of immune systems and osmotic regulation mechanisms is essential for the roughskin sculpin to cope with the challenges posed by exposure to diverse microbial communities and fluctuations in salinity during migration. Our work not only provides valuable resources for understanding the genetic basis of the roughskin sculpin’s adaptation to a unique migratory lifestyle, but may also offer important insights into how evolutionary radiation from marine to freshwater habitats has been achieved in marine-originated sculpins.

## ﻿Introduction

The evolutionary transitions from marine to freshwater habitat systems has occurred repeatedly throughout the evolutionary history of fish lineages (Bloom and Lovejoy 2017; Rabosky 2020). The abundant environmental resources and ecological niches in freshwater ecosystems provide extensive ecological opportunities, serving as significant driving factors for the evolutionary radiation of marine fishes (Gillespie et al. 2020). However, the transition from marine to freshwater habitats serves not only as an ecological change, but also present an extreme physiological challenge, as well (Lee and Bell 1999), for the interface between marine and freshwater habitats constitutes a formidable physiological barrier (e.g., salinity, oxygen, pH, pathogens), and requires significant behavioral, physiological, and morphological changes to overcome (Bloom and Lovejoy 2014). Consequently, many marine fishes have adopted a phased adaptive strategy during their evolution toward freshwater. Initially, individuals or lineages of a marine species venture into freshwater and transition into a diadromous species. Over time, some lineages of the diadromous species stop returning to marine systems, becoming completely freshwater species due to the ecological advantages of the new environment (Corush 2019). At present, multiple hypotheses have been proposed to explain the evolutionary significance of this diadromy, which is believed to serve as an intermediate stage during the marine and freshwater transition, and a crucial step in overcoming the physiological barriers (Gross et al. 1988). However, the evolutionary origins and the genetic basis underlying these unique diadromous adaptations, repeatedly evolved in fish lineages, remain poorly understood.

There are numerous taxa that serve as an intermediate during their lineage specific radiations from marine to freshwater ecosystem in the natural world, such as sticklebacks (Gasterosteidae), salmonids (Salmonidae), sculpins (Cottidae), and shads (Clupeidae) (McPhail 1994; Palkovacs et al. 2008). Among these, sculpins represent a lineage predominantly inhabiting the cold temperate regions of the Northern Hemisphere, comprising approximately 280 species (Nelson et al. 2016). While the majority of sculpins inhabit marine habitats, more than 60 species have been found in freshwater environments, including species from genera *Cottus*, *Trachidermus*, *Mesocottus*, and *Myoxocephalus*, as well as Baikal sculpins. These freshwater sculpins exhibit notable freshwater colonization behavior across all regions and are believed to have independently originated multiple times from different marine ancestors (Finnegan 2017). Some of these species exhibit diadromous migratory behavior and have adopted an intermediate strategies including catadromous and amphidromous life styles (Yokoyama and Goto 2005). Molecular phylogenetic analyses conducted on some *Cottus* species suggest that these freshwater sculpins may have undergone radiation in the northern regions of the Northern Hemisphere through two phases (Goto et al. 2015). The initial stage was the successful colonization of freshwater habitats in East Asia by species with a catadromous life history, and this lineage may have originated from a euryhaline marine sculpin ancestral species. However, due to physiological limitations related to osmotic regulation, their gametes, embryos, and larvae were not adapted to freshwater, necessitating their migration to the marine environments during reproduction. The second stage was the evolution of freshwater spawning sculpins, which have acquired the ability to spawn in freshwater habitats, subsequently spread to various freshwater habitats, and evolved amphidromous, lacustrine, and fluvial life styles (Goto et al. 2015). Therefore, these species within the family Cottidae that exhibit intermediate life strategies provide an ideal model for understanding the evolutionary origins of diadromy in fish lineages.

The roughskin sculpin (*Trachidermus fasciatus* Heckel, 1837) is one of the only two known catadromous sculpins, belonging to the family Cottidae, genus *Trachidermus* (Wang 1999). It is widely distributed in coastal waters of the Yellow Sea, Bohai Sea, and East China Sea in China, as well as in the rivers flowing into these marine areas, including those in the western and southern Korean Peninsula, and Kyushu Island in southern Japan (Onikura et al. 2002; Islam et al. 2007). During the breeding season, roughskin sculpin migrates to estuaries and spawns in shallow brackish waters, with the hatched larvae returning to rivers to grow and nourish. This unique catadromous life history indicates that the roughskin sculpin is likely one of the earliest species in Cottidae to colonize freshwater habitats, which is crucial for understanding the genetic basis of freshwater colonization by marine-origin sculpins. However, to date, the genetic basis of such diadromous adaptation in the roughskin sculpin remains largely unknown.

In the present study, we assembled a high-quality chromosome-level genome of roughskin sculpin. By comparing the genomic data of roughskin sculpin with those of 18 closely related teleost species, we aimed to elucidate the evolutionary origin of roughskin sculpin and identify the key genetic basis associated with its diadromous lifestyle. These results not only provide a valuable resource for understanding the genetic basis of the unique migratory lifestyle of the roughskin sculpin, but may also provide new insights into the mechanisms of the evolutionary radiation from marine to freshwater habitats in marine-originated sculpins.

## ﻿Materials and methods

### ﻿Sample DNA and RNA extraction

Roughskin sculpin (weighing between 41.5 and 69.0 grams) used in this study were obtained from the Yalu River in Dandong, Liaoning Province (39.99°N, 124.36°E). Muscle, brain, and gill tissues were collected and immediately stored in liquid nitrogen until DNA and RNA extraction. Genomic DNA was extracted from muscle tissue using the QIAGEN Genomic DNA Extraction Kit. Total RNA was extracted from brain and gill tissues using TRIzol reagent (TIANGEN), following the manufacturer’s protocol. The extracted nucleic acids were stored at -80 °C for subsequent analysis. All tissue collection and nucleic acid extraction procedures were conducted in accordance with the ethical guidelines provided by the Institutional Animal Care and Use Committee of Zhejiang Ocean University.

### ﻿Library construction and sequencing

To generate short-read data, genome DNA was fragmented into pieces with an average size of approximately 500 bp. An Illumina sequencing library was then generated and sequenced on the Illumina NovaSeq 6000 platform (Illumina, San Diego, CA, USA) in a 250 bp mode. For long-read sequencing, high-molecular-weight DNA was size-selected using the PippinHT system, repaired, and ligated with adapters using the SQK-LSK110 kit (Oxford Nanopore Technologies, UK). The library was quantified with a Qubit 3.0 fluorometer and sequenced on the Oxford Nanopore PromethION platform. Genomic DNA for the Hi-C library was extracted from roughskin sculpin, and sheared to a length of ~400 bp. The Hi-C library was then quantified and sequenced on the Illumina NovaSeq-6000 platform. These sequencing efforts provided a comprehensive dataset for subsequent genome assembly and analysis.

### ﻿Genome size estimation and assembly

After ﬁltering short reads for low-quality reads, defined as those with a Phred quality score below 20 for more than 40% of bases or containing more than five ambiguous bases (N), as well as duplicates and adapter sequences, using fastp (v. 0.23.1) (Chen et al. 2018), the clean data were utilized to estimate the genome size, heterozygosity, and repeat content of roughskin sculpin through k-mer (k = 17) analysis using Jellyfish (v. 2.3.0) (Marcais and Kingsford 2011) and GenomeScope software (Vurture et al.2017). Then Nanopore long reads were employed for the genome assembly, followed by removal of the low-quality reads and adapter sequences with NextCorrect module of NextDenovo (v. 2.4.0) (Hu et al. 2024). Redundant heterozygous regions were eliminated based on read depth using three rounds of Purge_dups (v. 1.2.5) with default parameters (Guan et al. 2020). Minimap2 (v. 2.24) was used as a sequence mapper for long-read polishing and redundancy removal (Li 2018).

For the generation of a high-quality chromosome-level assembly of the roughskin sculpin genome, Hi-C reads were aligned to the pre-assembled genome using Juicer (v. 1.6) (Durand et al. 2016b). Subsequently, 3d-dna (v. 180922) was executed to generate an input file for Juicerbox (Durand et al. 2016a; Dudchenko et al. 2017). This input file represents the assembly with contigs ordered and oriented in a candidate chromosome-level depiction. Using Juicerbox, we interactively updated the location and orientation of contigs and their delineation within chromosomes.

Finally, the completeness and accuracy of the genome assembly were assessed using BUSCO and reads mapping ratios. For BUSCO analysis, Benchmarking Universal Single-Copy Orthologs (BUSCO) software (v. 5.4.4) was employed to evaluate the genome assembly quality based on the actinopterygii_odb10 database (Simao et al. 2015). Additionally, for the analysis of read mapping ratios, sequencing reads from both Nanopore sequencing libraries and genomic Illumina short-insert libraries were aligned to the assembled genome using Minimap2 software and BWA software (v. 0.7.17) (Li and Durbin 2009), respectively.

### ﻿Genome annotation

Before gene annotation, we annotated repetitive sequences using *de novo* prediction and homology comparison methods. Initially, RepeatModeler (v. 2.0.1) was used to identify *de novo* repetitive sequences (Flynn et al. 2020). Subsequently, a combination of repetitive models from various sources was utilized in RepeatMasker (v. 4.1.5) to generate a soft-masked repetitive version of the assembled genome, which was then used for gene model annotation (Chen 2004).

For protein-coding gene annotation, we employed the following three methods: ab initio gene prediction, homology-based prediction, and RNA-seq-assisted prediction. First, for homology-based prediction, protein data of zebrafish was downloaded from GenBank and then used to predict gene structure using GeneWise (v. 2.4.1) (Birney et al. 2004). Second, for RNA-seq-assisted prediction, the RNA-seq data were aligned to the assembled genome using HISAT2 (v. 2.1.0), and then transcriptome assembly was de novo assembled by Trinity (v. 2.8.5) (Grabherr et al. 2011), which was used for gene structure prediction with the PASA pipeline (v. 2.4.1) (Haas et al. 2003). Third, for ab initio gene prediction, initial gene prediction results from PASA pipeline and GeneWise were integrated as the initial gene model, followed by gene structure prediction using AUGUSTUS (v. 3.3.3) (Stanke et al. 2008). Finally, EVM (v. 1.1.1) (Haas et al. 2008) and PASA pipeline (v. 2.4.1) were used to integrate and optimize prediction results from the three methods.

Additionally, we used Diamond software (v. 2.1.7) with an e-value threshold set at 1 × 10^-5^ to functionally annotate predicted genes by querying multiple public protein databases (KOG, Swiss-Prot, Pfam, EggNOG, and Nr) (Buchfink et al. 2015). InterproScan (v. 5.65) was also used to predict protein functions based on conservative protein domains by calling its built-in databases. Finally, we integrated annotation results from EggNOG and InterProScan to obtain gene ontology information and further annotated these gene sets using the Kyoto Encyclopedia of Genes and Genomes (KEGG) database (Kanehisa and Goto 2000).

### ﻿Phylogenetic tree construction and divergence time evaluation

To elucidate the phylogenetic position of the roughskin sculpin, we selected 18 closely related teleost species with published genome sequences (*Anoplopoma fimbria*, *Anarrhichthys ocellatus*, *Cottoperca gobio*, *Cyclopterus lumpus*, *Cynoglossus semilaevis*, *Danio rerio*, *Epinephelus lanceolatus*, *Gadus morhua*, *Gasterosteus aculeatus*, *Larimichthys crocea*, *Nibea albiflora*, *Pseudoliparis swirei*, *Pungitius pungitius*, *Seriola lalandi*, *Sebastes schlegelii*, *Sebastes umbrosus*, *Thamnaconus septentrionalis*, and *Xiphias gladius*), with roughskin sculpin genome sequence assembled in this study, to identify shared orthologs among these species. Except for *S. schlegelii*, which was downloaded from the CNSA (CNGB Nucleotide Sequence Archive) website, the whole genome sequence and annotation files for the other published species were downloaded from the NCBI database (Suppl. material 2: table S1). Self-to-self alignments were first conducted using Diamond (v. 2.1.7) BLASTp with an E-value threshold of 1 × 10^-5^. Hits with low quality (identity < 30% and coverage < 50%) were excluded. Orthologous groups were then identified using OrthoFinder (v. 2.5.5) with default settings, providing insights into the homology relationships among the selected species (Emms and Kelly 2019). Subsequently, orthogroups with protein sequence lengths > 6000 were filtered out using a Perl script. All the single-copy homologous genes shared by all 19 species ultimately obtained were concatenated into supergenes. Furthermore, Multiple sequence alignment of the target genes was performed using MAFFT (v. 7.407) to prepare for subsequent phylogenetic tree construction (Katoh and Standley 2013). The corresponding CDS alignments were obtained through reverse translation from protein alignments using pal2nal (v. 14.1) (Suyama et al. 2006). To improve phylogenetic resolution, we extracted the first two codon positions from CDS alignments to generate an alternative sequence set, as they are more conserved and less affected by synonymous substitutions than the third. IQ-TREE (v. 2.2.2.6) was utilized to infer the maximum likelihood tree with 1000 bootstrap replicates (Minh et al. 2020). Alignments of the complete protein sequences and the first two bases of each codon from all single-copy genes were used as input, with *D. rerio* designated as the outgroup species.

Divergence times among species were then estimated using a Bayesian relaxed molecular clock approach implemented in the MCMCtree program within the PAML package (v. 4.10.6) (Yang 2007). Fossil records of the divergence times of *D. rerio*-*G. morhua* (180.0–251.5 million years ago [Mya]), *G. morhua*-*S. lalandi* (128.0–165.0 Mya), *S. lalandi*-*L. crocea* (100.0–130.0 Mya), *L. crocea*-*E. lanceolatus* (83.2–150.9 Mya) and *E. lanceolatus*-*C. gobio* (61.7–89.9 Mya) from the TimeTree website (http://www.timetree.org) were used as the calibration times (Kumar et al. 2017).

### ﻿Estimation of relative evolutionary rates

The relative evolutionary rates of roughskin sculpin, in comparison with 18 other outgroup species, were computed using the protein sequences of all single-copy genes. Initially, all protein sequences were concatenated into a super-sequence and aligned using MAFFT (v. 7.407). The outgroup species and reference species were specified as *D. rerio* and *G. morhua*, respectively. Tajima’s relative rate test and a two-cluster analysis were executed using MEGA (v. 7.0.26) and LINTRE software, respectively (Tamura et al. 2007). In the Tajima’s relative rate test performed with MEGA, species-specific substitutions were calculated, and a higher number of species-specific substitutions indicated a faster evolutionary rate. In the LINTRE method, the evolutionary rate of each species was evaluated using Z statistics and the tpcv module with the parameter -distance=7.

### ﻿Estimation of gene family expansion and contraction

Using CAFE5 software (v. 5.1.0), we identified gene families in roughskin sculpin that have undergone expansion or contraction (Mendes et al. 2020). Utilizing information from the previously analyzed phylogenetic tree and divergence times, this analysis employed a probabilistic model to infer changes in gene family sizes. A significance threshold of *p*-value < 0.05 was set to indicate whether gene families had undergone significant changes. Subsequently, clusterProfiler (v. 4.10.0) was employed to conduct GO and KEGG pathway enrichment analysis on these expanded and contracted gene families, with significance determined by setting the *P*_adjust_ < 0.05 following Benjamini and Hochberg FDR correction (Wu et al. 2021).

### ﻿Detection of positive selection

In this study, we conducted an analysis of one-to-one single-copy orthologous genes identified in roughskin sculpin and 18 outgroup species to detect genes undergoing rapid evolution or positive selection. The dN/dS ratio was estimated using the CODEML program within the PAML package (v. 4.10.6) through *Branch models* and *Branch-site models*. Roughskin sculpin shares similar evolutionary characteristics in freshwater adaptation and migratory behavior with two other species from the family Gasterosteidae, namely the three-spined stickleback (*G. aculeatus*) and the nine-spined stickleback (*P. pungitius*) (Fang et al. 2020; Wang et al. 2020). Therefore, we designated these three teleosts as foreground branches, while four stenohaline marine teleosts within the suborder Cottoidei (*P. swirei*, *C. lumpus*, *A. ocellatus*, *A. fimbria*) were set as background branches. The one-ratio and two-ratio models in the *Branch models* were applied to detect ω values for the foreground and background branches. Genes were defined as rapidly evolving genes if they met the Chi-square test threshold (*p* < 0.05), with the ω value for foreground branches surpassing that for background branches and the ω for the foreground branches exceeding 0.8. Subsequently, while maintaining the species in the foreground and background branches constant, the A model (model=2, NSsites=2) of the Branch-site models was applied and compared with the corresponding null model through likelihood ratio test and Chi-square test to detect sites experiencing positive selection on the foreground branches, with a significance level set at *p* < 0.05. Additionally, Benjamini and Hochberg FDR correction was applied to eliminate false positives (Benjamini and Hochberg 1995). GO and KEGG enrichment analyses of these positively selected genes in the three foreground species were conducted using the clusterProfiler package, with a significance threshold of *p*-value < 0.05 to identify significantly over-represented GO terms and KEGG pathways.

## ﻿Results

### ﻿Assembly and characterization of the roughskin sculpin genome

**Figure 1.**
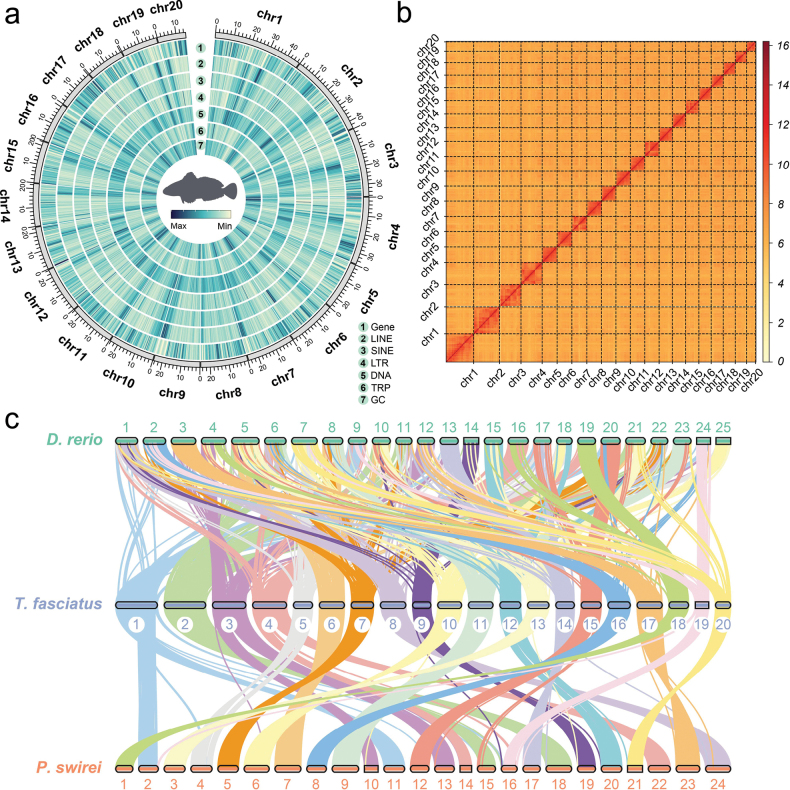
Genome assembly, annotation, and synteny analysis of the roughskin sculpin. a. Circos plot of genomic elements distribution in the roughskin sculpin, with a 400,000-bp window. From outer to inner ring are the distributions of protein-coding genes, long interspersed nuclear elements (LINEs), short interspersed nuclear elements (SINEs), long terminal repeats (LTRs), DNA transposons (DNA), tandem repeats (TRPs), and GC content; b. A heatmap of Hi-C interactions in the roughskin sculpin. The blocks along the diagonal represent the 20 pseudochromosomes, with the numbers at the bottom corresponding to specific chromosomes. The color bar illustrates contact density, ranging from yellow (low) to red (high); c. Chromosomal syntenic relationships between the roughskin sculpin and *D. rerio* and *P. swirei*, respectively. The numbers in the figure represent the chromosomes of each species, while each line represents the syntenic regions derived from LAST alignments.

We generated 87.58 Gb of Illumina short-insert size reads, providing a robust 163.12-fold coverage of the roughskin sculpin genome (Table 1, Suppl. material 2: table S2). The genome size was estimated to be ~485.12 Mb, with heterozygosity of 0.40% and repeat sequence content of 27.37% (Suppl. materials 1, 2: figs S1, S2, table S3). Finally, we constructed a high-quality chromosome-level assembly of the genome with 526.39 Mb with a scaffold N50 of 24.94 Mb (Table 2), and 98.05% of the assembly sequences were assigned to 20 pseudochromosomes (Fig. 1a, b, Suppl. material 2: table S4). The lengths of the genome’s chromosomes ranged from 15.11 to 47.09 Mb (Suppl. material 2: table S4). The 20 pseudochromosomes were easily distinguishable based on the heatmap, and the interaction signals around the diagonal were notably strong, indicative of the high quality of this genome assembly (Fig. 1b). In addition, the high completeness of the assembly (BUSCO: 98.5%, 3586/3640; Suppl. material 1: fig. S3, Table 3), the high mapping ratios of sequencing reads (98.63% for Illumina short reads, 99.31% for Nanopore long reads, and 99.22% for transcriptome Illumina reads; Suppl. material 2: tables S5, S6), and the strong synteny (Fig. 1c, Suppl. material 1: figs S4, S5, S6) all indicate that we have obtained a high-quality reference genome of roughskin sculpin.

**Table 1.** Statistics of the cleaned sequencing data for roughskin sculpin.

Nucleotide type	Sequencing strategy	Sequencing platform	Library size (bp)	Clean data (Gb)	Sequencing depth (x)
Genome	Nanopore	PromethION	Regular	53.61	99.86
Genome	Illumina	NovaSeq-6000	300	87.58	163.12
Genome	Hi-C	NovaSeq-6000	300	61.44	114.44
Transcriptome	Illumina	NovaSeq-6000	300	16.96	31.62

**Table 2.** Statistics of the assembled genome of roughskin sculpin based on the Nanopore and Hi-C data.

Term	NextDenovo		NextPolish		3D-DNA	
	size (bp)	Number	size (bp)	Number	size (bp)	Number
N50	20,687,790	11	20,755,963	11	24,942,754	8
N90	8,198,445	24	8,220,160	24	20,297,248	18
Max length (bp)	35,931,358		36,071,677		47,094,788	
Total length (bp)	540,971,557		536,852,755		526387896	
Total number	70		42		20	

**Table 3.** Roughskin sculpin genome assembly quality assessment based on BUSCO analysis.

Database	Actinopterygii_odb10	
	gene number	Percentage
Complete BUSCOs	3586	98.5
Complete and single-copy BUSCOs	3550	97.5
Complete and duplicated BUSCOs	36	1.0
Fragmented BUSCOs	13	0.4
Missing BUSCOs	41	1.1
Total BUSCO groups searched	3640	100.0

A total of 132.35 Mb of repetitive sequences, constituting 24.65% of the assembly, were identified in roughskin sculpin (Suppl. material 2: table S7). The repeat sequence content closely matched the value (27.37%) determined from the k-mer analysis (Suppl. material 2: table S3). Within these repeats, 3.80% were attributed to long interspersed elements (LINEs), 3.70% to simple repeats, 6.01% to DNA transposons, 0.92% to long terminal repeats (LTRs), and 0.87% to short interspersed elements (SINEs; Suppl. material 2: table S7). The prediction of protein-coding genes, which involved a combination of ab initio-based, homologue-based, and RNA-seq-based methods, resulted in the identification of 20,866 genes. These genes exhibited an average length of 13,017 bp, an average intron length of 10,282 bp, and an average exon length of 2,735 bp (Suppl. material 2: table S8). Fig. 1a comprehensively depicts the genome characteristics of roughskin sculpin. Of the predicted genes, 20,678 (99.10%) were successfully annotated through alignment with various public annotation databases, including KOG, KEGG, Pfam, Swiss-Prot, GO, InterPro, EggNOG, and Nr (Table 4). Moreover, ~95.80% (3489/3600) of complete BUSCOs specific to actinopterygians were detected in the annotated gene database, confirming the accuracy of the annotation process for the roughskin sculpin genome (Suppl. material 2: table S9).

**Table 4.** Functional annotation statistics of protein-coding genes in roughskin sculpin.

Annotation database	Annotated gene number	Percentage (%)
KOG	12,972	62.16
KEGG	14,822	71.03
Pfam	19,005	91.08
SwissProt	19,459	93.25
GO	19,500	93.45
InterProScan	20,134	96.49
EggNOG	20,467	98.08
Nr	20,612	98.78
All annotated genes	20,678	99.10
All Predicted genes	20,866	100.00

### ﻿Comparative genomics and phylogenetic analysis

**Figure 2.**
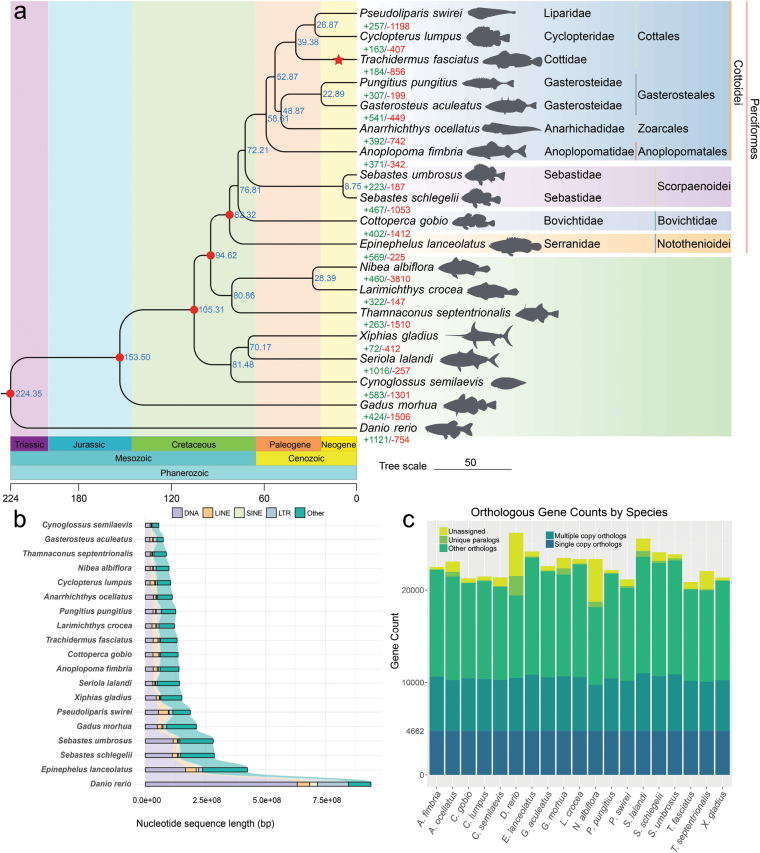
Phylogenetic relationships, repeat sequence statistics, and orthologous gene counts of roughskin sculpin and closely related species. a. Phylogenetic relationships and divergence times between roughskin sculpin and 18 outgroup species. The position of roughskin sculpin on the evolutionary tree is highlighted with a red star. Blue numbers at each node represent divergence times between species, while the red circle indicates the fossil record used for calibration at that node. Green and red numbers indicate gene families that have expanded and contracted at each node, respectively. The geological periods and ages (Million years ago, Mya) are indicated along the bottom; b. Repeat sequence sizes and transposable element (TE) statistics of roughskin sculpin and other 18 teleost species. The figure displays the sizes of each element type (including DNA elements, LINEs, SINEs, LTRs, and other repeat sequence regions) within each species; c. Statistics of the number of genes with various types of orthologs in roughskin sculpin and 18 other teleost species.

A comparative genomic analysis between the roughskin sculpin genome and those of from 18 other teleost species, including closely related species within the suborder Scorpaenoidei and the order Perciformes, revealed a total of 23,405 gene orthogroups, of which 8,402 were shared across all 19 species (Suppl. material 2: table S10). Specifically, the 20,137 genes identified in roughskin sculpin were clustered into 17,660 gene orthogroups, including 22 unique gene orthogroups containing 59 genes. In total, we obtained 4,662 single-copy orthologous genes (Fig. 2c, Suppl. material 2: table S10). By combining the roughskin sculpin genome with those of 18 outgroup species, we constructed maximum likelihood (ML) phylogenetic trees among these species using concatenated sequences derived from the amino acids (Q.plant+F+I+R8 model) and the first two bases of codons (GTR+F+I+R5 model) of single-copy orthologous genes. The topologies of the phylogenetic trees reconstructed using the two methods described above were consistent. Our results indicated that roughskin sculpin, along with two other Cottales species, *P. swirei* (Liparidae) and *C. lumpus* (Cyclopteridae), formed one clade. The reconstructed phylogenetic relationships within the suborder Cottoidei are consistent with the hypothesis that roughskin sculpin may have originated from a marine ancestral species.

**Figure 3.**
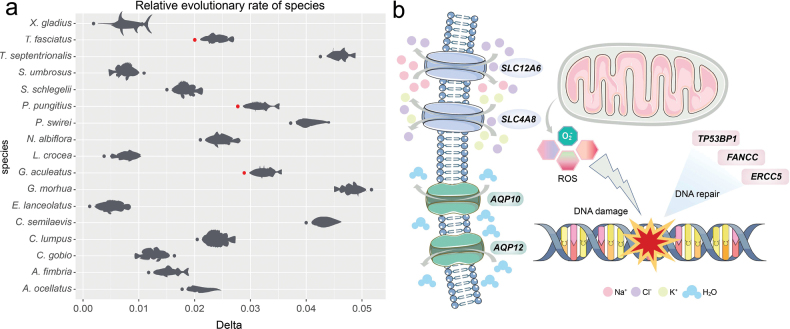
Relative evolutionary rate of roughskin sculpin and genes under positive selection. a. Relative evolutionary rates between roughskin sculpin and 18 other outgroup species, with *D. rerio* designated as the outgroup and *S. lalandi* serving as the reference species. Roughskin sculpin, with two other species exhibiting diadromous migratory habits, *G. aculeatus* and *P. pungitius*, are highlighted in red; b. Positive selection genes associated with DNA repair and osmotic regulation in roughskin sculpin, which may help mitigate the negative effects of prolonged metabolic activity and salinity changes during migration.

Such an extreme habitat shift may impose stronger selective pressure on roughskin sculpin, potentially leading to a faster evolutionary rate. To evaluate this hypothesis, we calculated the relative evolutionary rates of roughskin sculpin and 18 other outgroup species using single-copy orthologous genes. The analysis revealed that roughskin sculpin exhibited a relatively high evolutionary rate (Fig. 3a, Suppl. material 2: table S11), suggesting it may have experienced intense adaptive evolution, although other factors such as limited population size and rapid genetic drift cannot be ruled out (Kimura and Ota 1971). Using fossil calibrations, we estimated that the Cottidae branch to which roughskin sculpin belongs diverged from the common ancestor of the Liparidae and Cyclopteridae in the Middle Eocene ~39.38 Mya (Fig. 2a). This divergence time was generally consistent with the estimate of the divergence time between the Liparidae and Cottidae families (40.8 Mya) derived from whole-genome analyses by Xue et al. (Xue et al. 2022).

### ﻿Genetic alterations correlated with diadromous migration adaptations

**Figure 4.**
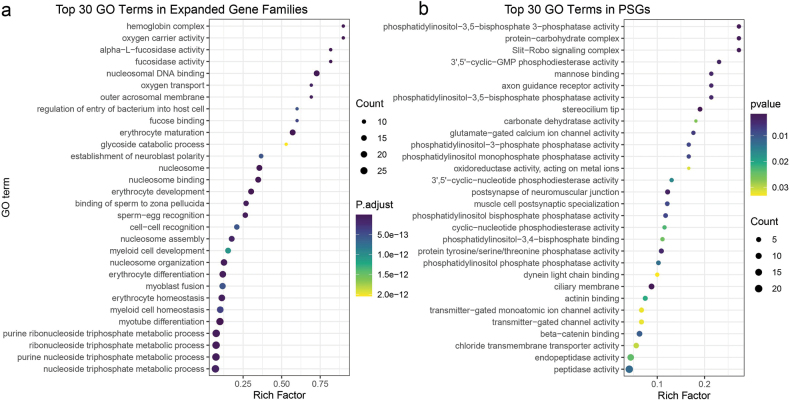
GO enrichment analysis of expanded gene family genes and PSGs. a. Top 30 GO enrichment results of genes in the expanded gene family of roughskin sculpin, with the horizontal axis representing the enrichment factor and the vertical axis representing the enriched GO terms; b. Top 30 GO enrichment results of genes under positive selection in roughskin sculpin, with *G. aculeatus* and *P. pungitius* as foreground branches.

To investigate this hypothesis, we conducted a comparative genomic analysis involving roughskin sculpin and 18 other outgroup species, with the goal of identifying gene that underwent significant changes within the roughskin sculpin genome during evolution. The results revealed that in roughskin sculpin, 184 gene families underwent expansion, while 856 gene families experienced contraction (*p* < 0.05) (Fig. 2a). The expanded gene families of roughskin sculpin were significantly enriched in multiple GO terms and KEGG pathways, which mainly included nucleosomal DNA binding (GO:0031492, *p*-value = 2.31 × 10^-27^), erythrocyte maturation (GO:0043249, *p*-value = 2.27 × 10^-25^), oxygen carrier activity (GO:0005344, *p*-value = 2.40 × 10^-17^), and immune system (ko05322, *p*-value = 2.80 × 10^-18^) that were mainly related to nucleosomes, oxygen transport and immune response (Fig. 4a, Suppl. materials 1, 2: fig. S7, tables S12, S13). Conversely, the top contracted gene families were significantly enriched in nucleosome organization (GO:0034728, *p*-value = 3.89 × 10^-13^), nucleotide binding (GO:0019001, *p*-value = 5.87 × 10^-12^), and gap junction (ko04540, *p*-value = 1.88 × 10^-1 4^) that were associated with histones and tight junctions (Suppl. materials 1, 2: figs S8, S9, tables S14, S15). The significant expansion of these gene families related to oxygen transport and immune response may indicate their important roles in the migration process of roughskin sculpin.

We also identified a total of 477 genes that have undergone positive selection and two rapidly evolving genes by treating roughskin sculpin, with two diadromous species of *P. pungitius* and *G. aculeatus* from Gasterosteidae, as a foreground clade using branch-site and branch models of the codeml program (Suppl. material 2: table S16). Further functional annotation through GO and KEGG enrichment analysis has revealed several pathways associated with the adaptations of their diadromous migratory lifestyles. These pathways include chloride ion transmembrane transporter activity (*SLC4A8*, *SLC12A6*, and *SLC26A10*) (Mount et al. 1999; Mount and Romero 2004; Leviel et al. 2010), DNA damage and repair (*FANCC*, *TP53BP1*, and *ERCC5*) (Pang et al. 2001; Chapman et al. 2012; Soltys et al. 2013), and innate immune and inflammatory responses (*CL-11*, *LRCH4*, *NFKBID*, and *CARD8*) (Hansen et al. 2010; Arnold et al. 2012; Mao et al. 2018; Aloor et al. 2019) (Fig. 4b, Suppl. material 2: tables S17, S18). Evidence of positive selection in genes related to chloride ion transmembrane transport, DNA repair, and innate immunity may indicate that these diadromous species has undergone adaptive remodeling in osmoregulation, circulatory, and immune system to cope with challenges such as salinity fluctuations, oxygen demand, and pathogens from different microbial communities encountered during migration.

## ﻿Discussion

### ﻿Characteristics of genomic sequences in the roughskin sculpin

By integrating data from multiple sequencing technologies and utilizing high-resolution chromosomes linkage mapping strategy, we successfully generated a chromosome-level genome assembly of roughskin sculpin with a genomic size of 526.39 Mb, which was anchored to 20 pseudo-chromosomes. The contig N50 and chromosome N50 of roughskin sculpin genome are 20.76 Mb and 24.94 Mb, respectively, indicating extremely high continuity in the assembled sequences. The completeness and accuracy of roughskin sculpin genome were evaluated using BUSCO based on the actinopterygii_odb10 dataset, achieving scores of 98.5% and 95.8%, respectively. In addition, up to 99.1% of the 20,866 predicted genes have been functionally annotated in multiple public gene databases. These data demonstrate that our genome assembly and annotation are complete and of good quality. However, it is noteworthy that the genome of roughskin sculpin is relatively small compared to other known bony fish, which may suggest that it possesses characteristics of a compact genome. Previous studies have shown that repetitive sequences, particularly transposable elements (TEs), are not only a major component of vertebrate genomes but also play a decisive role in genome size expansion and the evolution of genomic structural diversity (Sotero-Caio et al. 2017). Therefore, to explore the characteristics of TEs in roughskin sculpin and their possible contribution to its small genome size, we conducted repeat sequence annotation and performed comparative analysis with TEs data from other published species (Fig. 2b, Suppl. material 2: table S19). The results of the repeat sequence annotation revealed that Tc/mariner, hAT, L2, and Gypsy are the predominant TE superfamilies in the roughskin sculpin genome (Suppl. material 2: table S7), consistent with the findings of Shao et al. (2019) on TE diversity in fish. Additionally, the total repeat sequence content in the genome of roughskin sculpin is relatively low, accounting for approximately one-quarter (24.65%/129.75 Mb) of the assembled genome. This proportion is very similar to that of the closely related nine-spined stickleback (23.22%/121.02 Mb), but much lower than the medaka (33.70%/236.28 Mb) and zebrafish (56.49%/773.70 Mb) where TEs have significantly expanded (Suppl. material 2: table S19) (Gao et al. 2016). While broader comparisons across more species are needed to fully assess lineage-wide trends, these findings suggest that the relatively low TE content may contribute to the compact genome architecture observed in roughskin sculpin.

The compactness of roughskin sculpin genome is evident not only in its genome size but also in its chromosome number. Our genome assembly results indicate that the haploid chromosome count of roughskin sculpin is only 20, which is lower than the 24 found in most species within Perciformes (Nirchio et al. 2014). This finding suggests that roughskin sculpin genome may have undergone chromosomal fusion events. To verify this hypothesis, we conducted a synteny analysis between roughskin sculpin and *P. swirei*, a species within the same suborder Cottales that retains the ancestral chromosome number (*n* = 24), making it a suitable reference for identifying potential fusion events. The results revealed a high degree of synteny between the two species, but the pseudochromosomes 1, 2, 3, and 4 of roughskin sculpin may have undergone chromosomal fusions relative to *P. swirei* (Fig. 1c, Suppl. material 1: fig. S5). In addition, we examined two phylogenetically close diadromous relatives of roughskin sculpin, *G. aculeatus* and *P. pungitius*, both of which have a haploid chromosome number of 21 (Ocalewicz et al. 2011), have also undergone chromosomal fusion, and exhibit a diadromous lifestyle similar to that of roughskin sculpin. By comparing these species, we aimed to further explore whether such chromosomal rearrangements also constitute the genetic basis of diadromous migration adaptations in roughskin sculpin. Previous studies have shown that these two species also experienced chromosome fusion events, which may have created low-recombination regions due to suppressed crossover activity near the fusion breakpoints. Such structural changes can alter the recombination landscape and limit genetic exchange in those regions. During the repeated glacial and interglacial periods of the Pleistocene, these low-recombination regions may have promoted the accumulation of adaptive alleles and formed adaptive clusters that may impede the gene flow, thereby helping them gradually adapt to freshwater environments (Liu et al. 2022). Therefore, we hypothesize that chromosomal fusions in roughskin sculpin may also help resist gene flow with its marine ancestor species, providing a genomic foundation for its adaptation to freshwater environments and the evolution of diadromous lifestyle. However, this hypothesis requires further investigation of the genes at the chromosomal fusion sites in roughskin sculpin to validate it.

### ﻿Genetic origin of the diadroumous lifestyle in roughskin sculpin

There have been many studies on the evolutionary origin of the diadromous lifestyle in fish lineages. Multiple hypotheses have been proposed to explain the evolutionary origin of diadromy. Among them, the “productivity hypothesis” suggests that uneven resource distribution across different habitats may promote species to choose environments with higher productivity during specific life stages, and this resource-driven migratory behavior may eventually form a stable life history trait over time (Gross et al. 1988). The “safe-site hypothesis” posits that freshwater habitats provide a refuge for marine eggs and larvae from marine predators, and the adaptive advantages provided by migrating to freshwater habitats for reproduction will drive the evolutionary origin of diadromy. Additionally, the paleogeography and geological history of certain regions may also have an important impact on the evolution of diadromy. For example, northern latitudes typically have a high proportion of diadromous lineages due to repeated glacial advances and retreats (McDowall 2008). Our phylogenetic analysis and previous research results seem to be consistent with the glacial-driven origin hypothesis. Firstly, the phylogenetic relationships we reconstructed suggest that roughskin sculpin may origin from marine sculpins. It clusters with *C. lumpus* and *P. swirei* and these are paraphyletic with species of the families Bovichtidae and Serranidae within the order Perciformes. This aligns with the current classification system that places the families Cottidae, Cyclopteridae, and Liparidae within the suborder Cottales, and further supports the validity of the new classification method of Cottoidei and Scorpaenoidei as suborders within Perciformes (Betancur et al. 2017). The phylogenetic tree indicates that the closest relatives of roughskin sculpin, *C. lumpus* and *P. swirei*, are both marine stenohaline species, suggesting that roughskin sculpin may have originally derived from a marine ancestral species and then gradually transitioned to a freshwater habitat. Secondly, the calibrated divergent time of roughskin sculpin seems to support a scenario of their glacial-driven origin. Although our phylogenetic analysis cannot give an exact time of roughskin sculpin’s origin from the ancestors, partly due to the lack of genomic information from more closely related Cottidae species. However, previous phylogenetic analysis of Cottidae species based on mitochondrial DNA data has indicated that the evolutionary radiation of freshwater sculpins in East Asia occurred in the Pliocene-Pleistocene (Yokoyama and Goto 2005; Goto et al. 2015). The global climate during the Pleistocene was characterized by dramatic temperature fluctuations, with ice sheets periodically advancing and receding, resulting in rapid changes in species distributions (Hewitt 2000; Yokoyama and Goto 2005). One of the major consequences of the Pleistocene glaciation in East Asia was a significant change in sea level. As the sea level dropped significantly during the glacial period, parts of the shallow marine waters of the East Asian continent and the Korean Peninsula were exposed, forming a large number of inland lakes connected to the sea (Qian and Ricklefs 2000; Fu and Wen 2023). These newly formed freshwater lakes flowing to the sea may have provided refuges during the glacial period, and thereby facilitating the freshwater colonization and hence the evolution of a diadromous lifestyle in the roughskin sculpin.

### ﻿Genomic signatures associated with the diadromous adaptations in the roughskin sculpin

However, such an evolutionary scenario may imply stronger selective pressure imposed on the roughskin sculpin, potentially leading to substantial genetic remodeling associated with adaptation to a transitional habitat from marine to freshwater environments. Indeed, our adaptive evolution analysis indicated that multiple gene families associated with oxygen transport and DNA repair have undergone significant alterations in the genome of roughskin sculpin. Some gene families closely associated with oxygen transport have undergone significant expansion in roughskin sculpin, including hemoglobin subunit alpha 1 (*HBA1*) and oxygen transporting hemoglobin embryonic a subunit (*hbae*) (Suppl. material 2: table S12). We inferred that the expansion of the *hbae* gene may ensure sufficient oxygen supply during the embryonic and juvenile stages, thereby promoting tissue development, while the expansion of the *HBA1* gene may enhance the oxygen-carrying capacity of hemoglobin, enabling roughskin sculpin to maintain metabolic function more effectively during long-distance migration (Wells 2009; Kelly et al. 2020). Although both genes are involved in oxygen transport, their distinct temporal expression patterns suggest stage-specific roles that together support the diadromous migration adaptations in the roughskin sculpin. Additionally, several genes related to DNA repair, including *FANCC* (PSG, *p*-value = 9.98 × 10^−10^), *TP53BP1* (PSG, *p*-value = 1.83 × 10^−6^), and *ERCC5* (PSG, *p*-value = 4.84 × 10^−3^), have also undergone positive selection in roughskin sculpin (Fig. 3b, Suppl. material 2: table S20). These genes play a critical role in the recognition and repair of damaged DNA (Pang et al. 2001; Chapman et al. 2012; Soltys et al. 2013). During migration, prolonged periods of intense metabolic activity, such as sustained swimming and increased energy consumption, lead to elevated production of reactive oxygen species (ROS), which can directly damage DNA and lead to cell senescence or death (Breitenbach et al. 2014). ROS-mediated DNA damage can be repaired through multiple DNA repair pathways, thereby maintaining genomic integrity and stability (Morita et al. 2010). Therefore, the positive selection of genes related to DNA repair in roughskin sculpin may reflect the necessity of enhancing DNA repair capacity to mitigate the negative effects associated with long-distance migration.

In addition to the metabolic pressures associated with long-distance migration, roughskin sculpin also needs to cope with environmental pressures derived from diverged pathogens inhibiting both marine and freshwater habitats (Logares et al. 2009). We found that multiple genes related to immune response have undergone expansion and adaptive changes in roughskin sculpin, possibly indicated their role in response to the diverse pathogens under different salinity gradients (Lokesh and Kiron 2016). The expanded gene families include members of the tripartite motif (TRIM) gene family, such as *TRIM21* and *TRIM39*, and GTPase of the immunity-associated protein (GIMAP) gene family, including *GIMAP8* and *GIMAP9*, and heat shock protein 70 (*hsp70*) (Suppl. material 2: tables S12, S13). Most members of the TRIM protein family possess E3 ubiquitin ligase activity and play roles in cellular processes including autophagy, apoptosis, and innate immunity (Hatakeyama 2017). The *Gimap* gene family has been shown to be most highly expressed in immune system cells in vertebrates and is a crucial component for T cell survival and development (Nitta and Takahama 2007). Hsp70 acts as a molecular chaperone that binds non-protein molecules with exposed hydrophobic residues, such as LPS, lipoproteins, and flagelin, playing a critical role in antigen presentation, cross-presentation, and the activation of macrophages and lymphocytes (Tsan and Gao 2009). In addition, several immune-related genes, such as *Nfkbid* (PSG, *p*-value = 3.81 × 10^−7^), *CL-11* (PSG, *p*-value = 7.51 × 10^−5^), *CARD8* (PSG, *p*-value = 8.14 × 10^−4^), and *Lrch4* (PSG, *p*-value = 1.21 × 10^−3^) were observed to be positively selected in roughskin sculpin (Suppl. material 2: table S16). These genes are involved in the complex regulation of the immune system, playing crucial roles in immune responses and inflammation regulation. Thus, the significant alterations in these genes may provide roughskin sculpin with enhanced immune defenses against various pathogens observed in different salinity habitats. However, this hypothesis needs further validation by comparing the genomes of more stenohaline teleosts to determine whether the alterations of these genes are universally present in diadromous fish.

In addition to pathogens and metabolic pressures, roughskin sculpin also needs to cope with frequent osmotic challenges along salinity gradients during their diadromous migration. Our positive selection analysis revealed that multiple genes associated with osmoregulation have undergone positive selection in roughskin sculpin. These genes include *SLC4A8* (PSG, *p*-value = 5.45 × 10^−5^), *SLC12A6* (PSG, *p*-value = 7.68 × 10^−9^), *AQP10* (PSG, *p*-value = 5.11 × 10^−11^), and *AQP12* (PSG, *p*-value = 1.65 × 10^−6^) (Fig. 3b, Suppl. material 2: table S16). Among them, the proteins encoded by *SLC4A8* and *SLC12A6* are closely involved in mediating the exchange of Na^+^ or K^+^ with Cl^−^ to maintain Cl^−^ homeostasis, playing important roles in cellular osmoregulation (Hebert et al. 2004; Romero et al. 2004). The *SLC4A8* gene encodes a Na^+^-driven Cl^−^/HCO_3_^−^ exchanger (NDCBE), which is an electroneutral transporter typically mediating the exchange of 1 Na^+^ and 2 HCO_3_^−^ for 1 Cl^−^ (Grichtchenko et al. 2001). Studies have shown that NDCBE plays a critical role in the net electroneutral Na^+^ reabsorption and osmotic regulation in the apical membrane of mouse collecting ducts (Leviel et al. 2010). The expression of the *SLC12A6* gene is activated by cell swelling under hypotonic conditions, with the encoded KCC3 promoting the efflux of K^+^ and Cl^−^, thereby preventing excessive cell swelling and contributing to osmotic stability (Adragna et al. 2015). Additionally, the *AQP10* and *AQP12* genes encode an aquaglyceroporin and a superaquaporin, respectively (Nozaki et al. 2008). AQP10 was initially identified in the apical membrane of the human small intestine and is believed to be involved in the transport of water and small solutes (Hatakeyama et al. 2001). Silencing of this gene has been shown to significantly reduce the permeability of glycerol and water in differentiated human adipocytes (Laforenza et al. 2013). AQP12 is selectively expressed in the pancreas of mammals and is believed to participate in the regulation of pancreatic secretion (Ishibashi 2009). The expression levels of these two genes are closely related to environmental salinity. Previous studies have reported significant alterations in the tissue distribution patterns of these genes in both medaka and roughskin sculpin after acclimation to different salinity conditions, indicating their important role in the adaptation of fish to different salinity conditions (Kim et al. 2014; Ma et al. 2020). The significant alterations in these osmoregulation genes may assist roughskin sculpin in regulating ion and water balance under hypertonic and hypotonic conditions, maintaining osmotic homeostasis and acid-base balance, thereby facilitating their adaptations to osmotic changes during the catadromous migration.

## ﻿Conclusions

In this study, we generated a high-quality chromosome-level genome assembly for roughskin sculpin, which comprised 20 pseudochromosomes with a total size of 526.39 Mb and a scaffold N50 of 24.94 Mb. The phylogenetic tree supports that the order Scorpaeniformes is not a valid monophyletic group and should be integrated into the order Perciformes. Accordingly, sculpins are now considered to originate from the suborder Cottoidei within Perciformes, rather than from the previously recognized Scorpaeniformes. The comparative analysis indicated that multiple gene families related to oxygen transport and immune response have expanded in the roughskin sculpin genome, and many genes related to DNA repair, immune response and osmoregulation have undergone significant alterations in roughskin sculpin. Our findings may not only help reveal the genetic basis underlying the roughskin sculpin’s adaptation to its unique catadromous lifestyle, but also contribute to enhancing our understanding of how evolutionary radiation from marine to freshwater habitats has been achieved in many marine-originated teleosts. The assembled genome and annotated data generated in this study will also serve as valuable resources for future research on teleost systematics and population genomics.

## ﻿Additional information

### Use of AI

No use of AI was reported.

### Data availability

All of the data that support the findings of this study are available in the main text or Supplementary Information.

## Supplementary materials

Supplementary material 1 Supplementary images Data type docx https://binary.pensoft.net/file/1447168This dataset is made available under the Open Database License (http://opendatacommons.org/licenses/odbl/1.0/). The Open Database License (ODbL) is a license agreement intended to allow users to freely share, modify, and use this Dataset while maintaining this same freedom for others, provided that the original source and author(s) are credited.Tianwei Liu, Kun Huang, An Xu, Zhenming Lü, Li Gong, Jing Liu, Ming Tang, Liqin Liu

Supplementary material 2 Supplementary tables Data type xlsx https://binary.pensoft.net/file/1447169This dataset is made available under the Open Database License (http://opendatacommons.org/licenses/odbl/1.0/). The Open Database License (ODbL) is a license agreement intended to allow users to freely share, modify, and use this Dataset while maintaining this same freedom for others, provided that the original source and author(s) are credited.Tianwei Liu, Kun Huang, An Xu, Zhenming Lü, Li Gong, Jing Liu, Ming Tang, Liqin Liu
