# Supplementary material for: ﻿Genome sequencing provides novel insights into diadromous migration adaptations in the roughskin sculpin, Trachidermus fasciatus (Scorpaeniformes, Cottidae)
Source: Zookeys. 2025 Oct 23;1256:293–316. doi: 10.3897/zookeys.1256.153772 (PMC12576477; doi:10.3897/zookeys.1256.153772)
Supplement: Supplementary material 1 — Supplementary images [file zookeys-1256-293_article-153772__-s001.docx]

Supplementary Figure


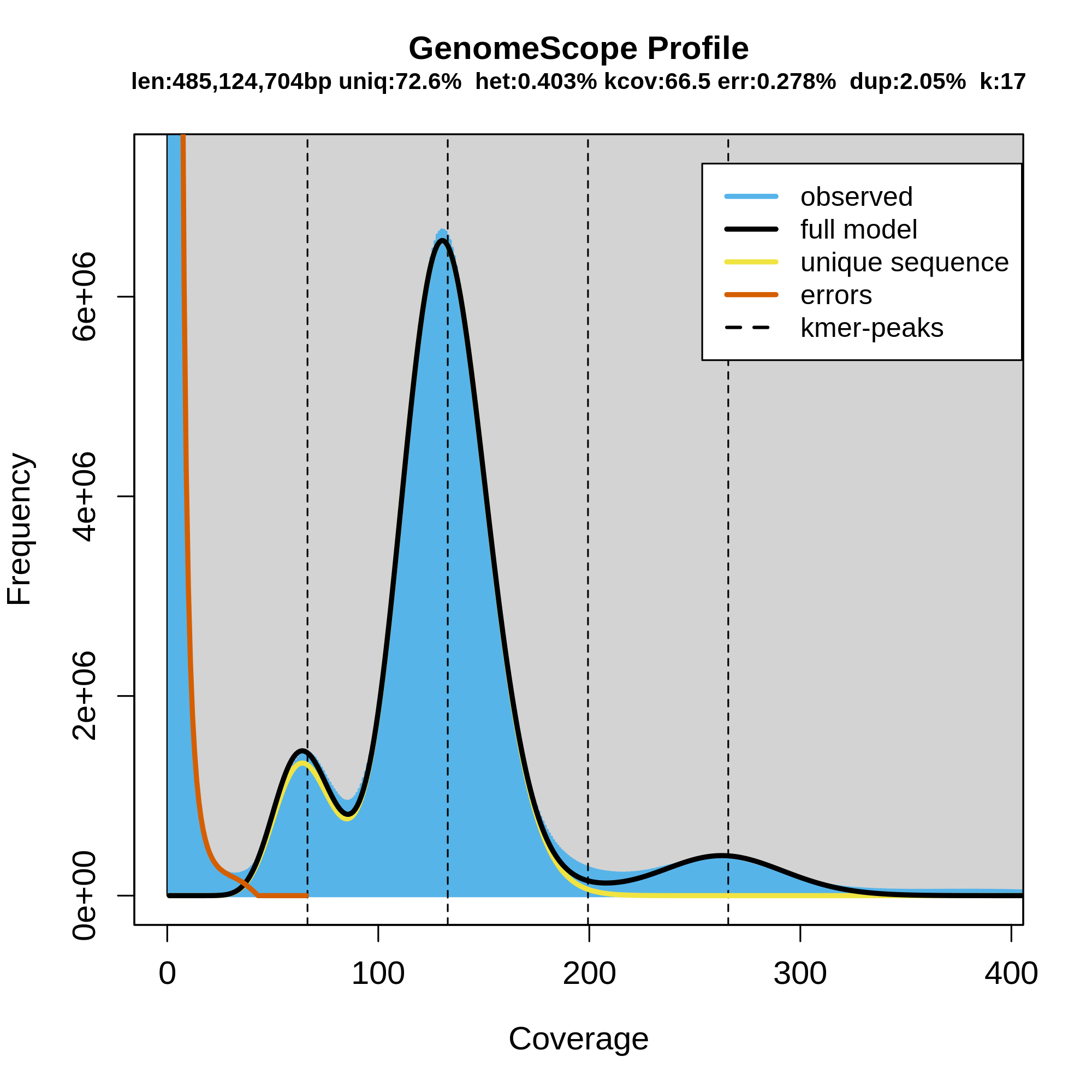


Figure. S1. K-mer analysis for roughskin sculpin genome size estimation, illustrating the distribution of 17-mer frequencies. The X-axis represents the K-mer depth, the Y-axis represents the K-mer frequency at each depth, and the first and second peaks correspond to the heterozygous and homozygous peaks, respectively.


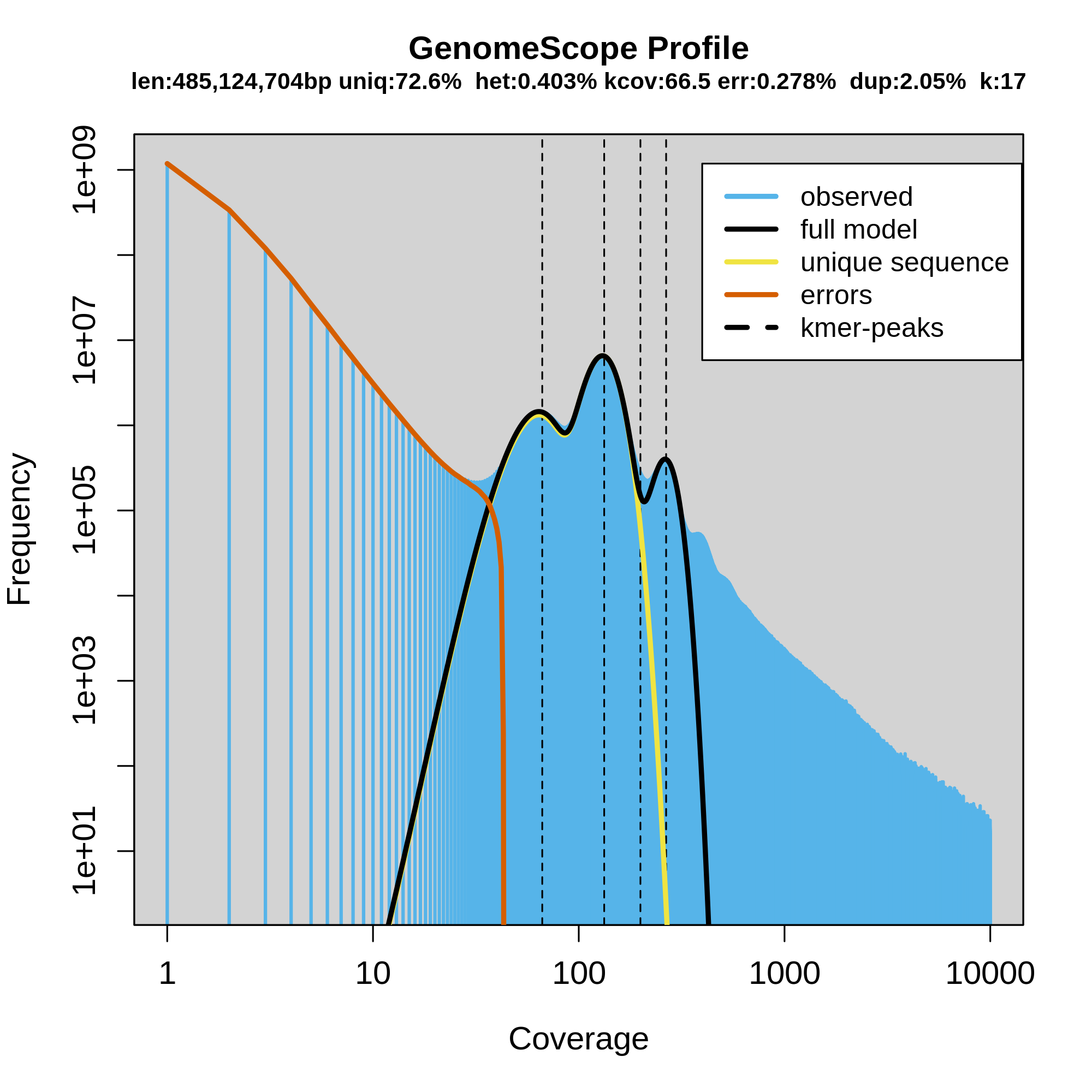


Figure. S2. The GenomeScope profile for roughskin sculpin was generated using results from Jellyfish v2.2.6 with a k-mer size of 17 as input for GenomeScope v1.0. The estimated heterozygosity of roughskin sculpin is 0.403%, and the estimated genome size is 485 Mb.


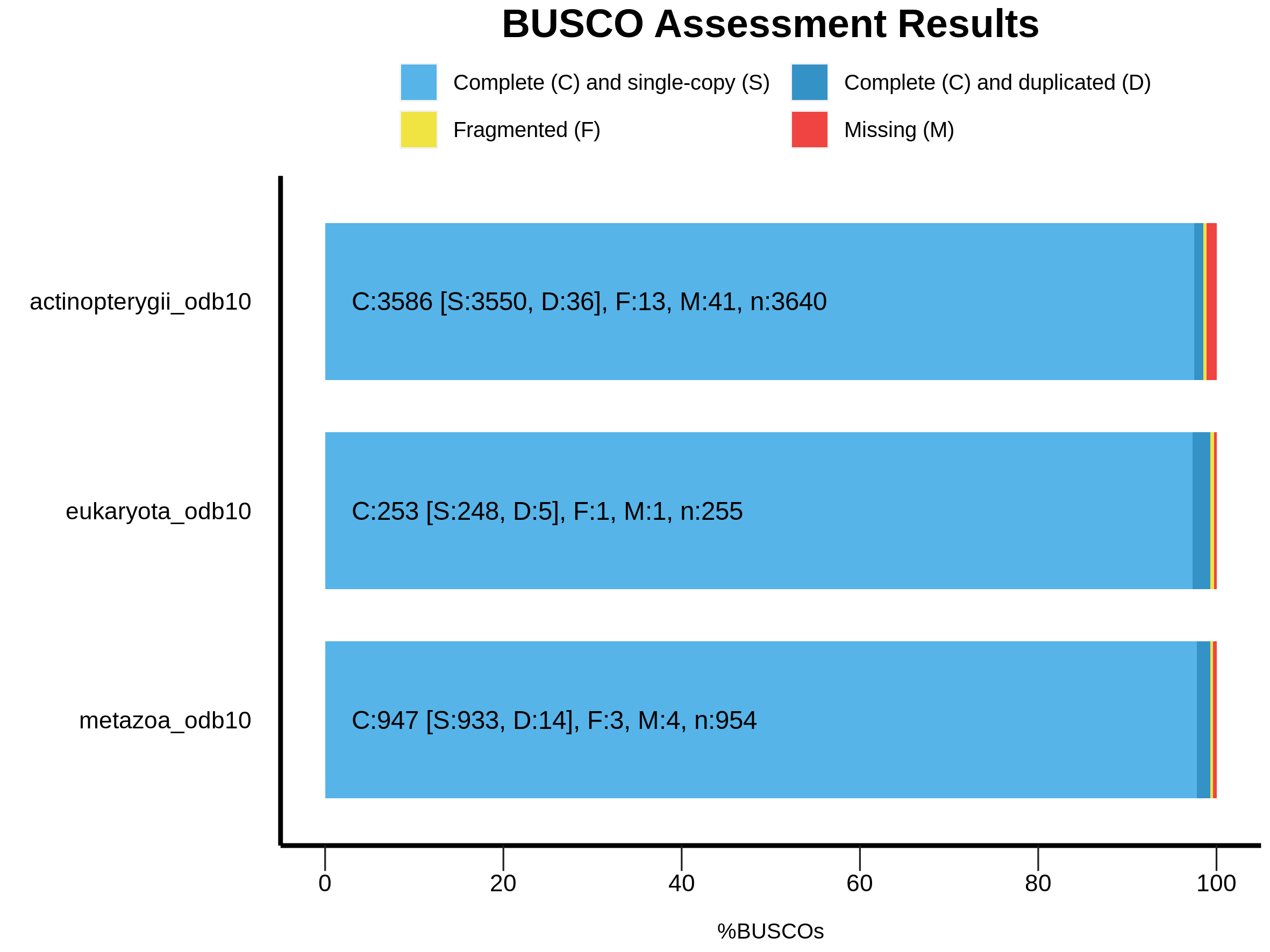


Figure. S3. The BUSCO software was employed to evaluate the genome assembly of roughskin sculpin, using the Actinopterygii, eukaryotic, and metazoan databases, respectively.


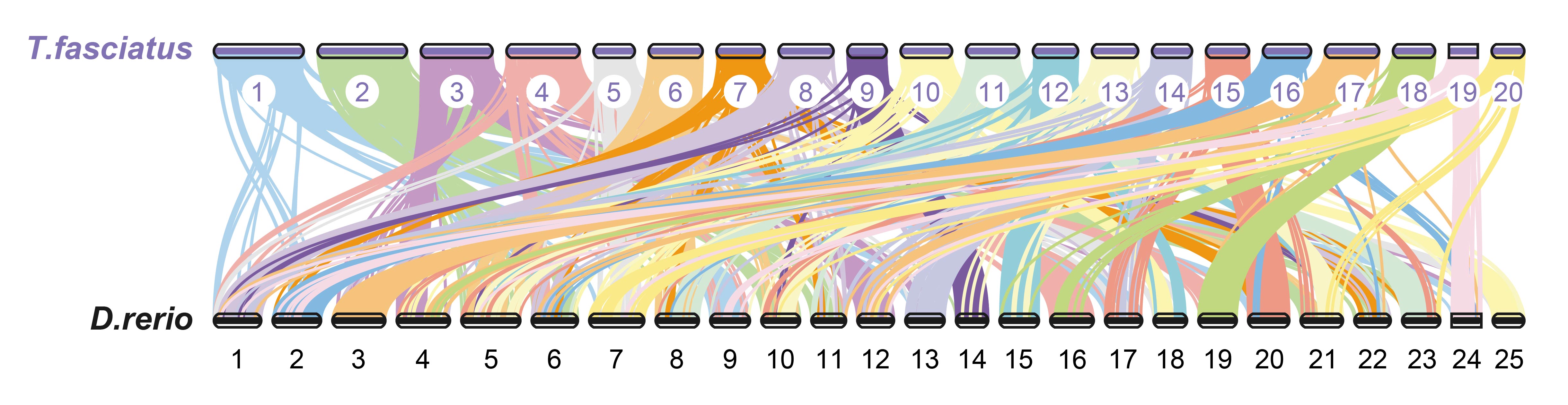


Figure. **S4.** Chromosomal syntenic relationships between the roughskin sculpin and *D. rerio*. The numbers in the figure represent the chromosomes of each species, while each line represents the syntenic regions derived from LAST alignments.


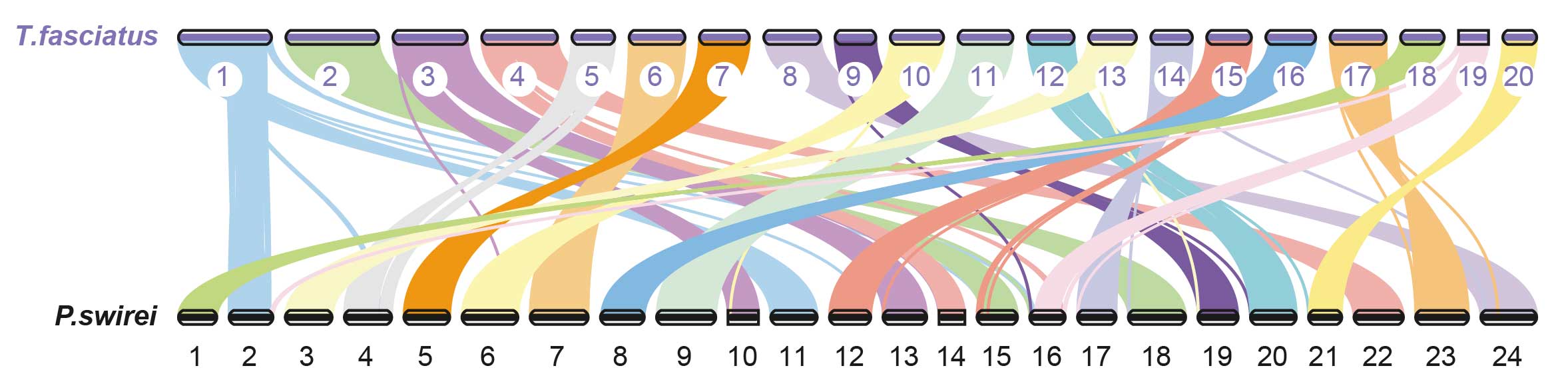


Figure. **S5.** Chromosomal syntenic relationships between the roughskin sculpin and *P. swirei*. The numbers in the figure represent the chromosomes of each species, while each line represents the syntenic regions derived from LAST alignments.


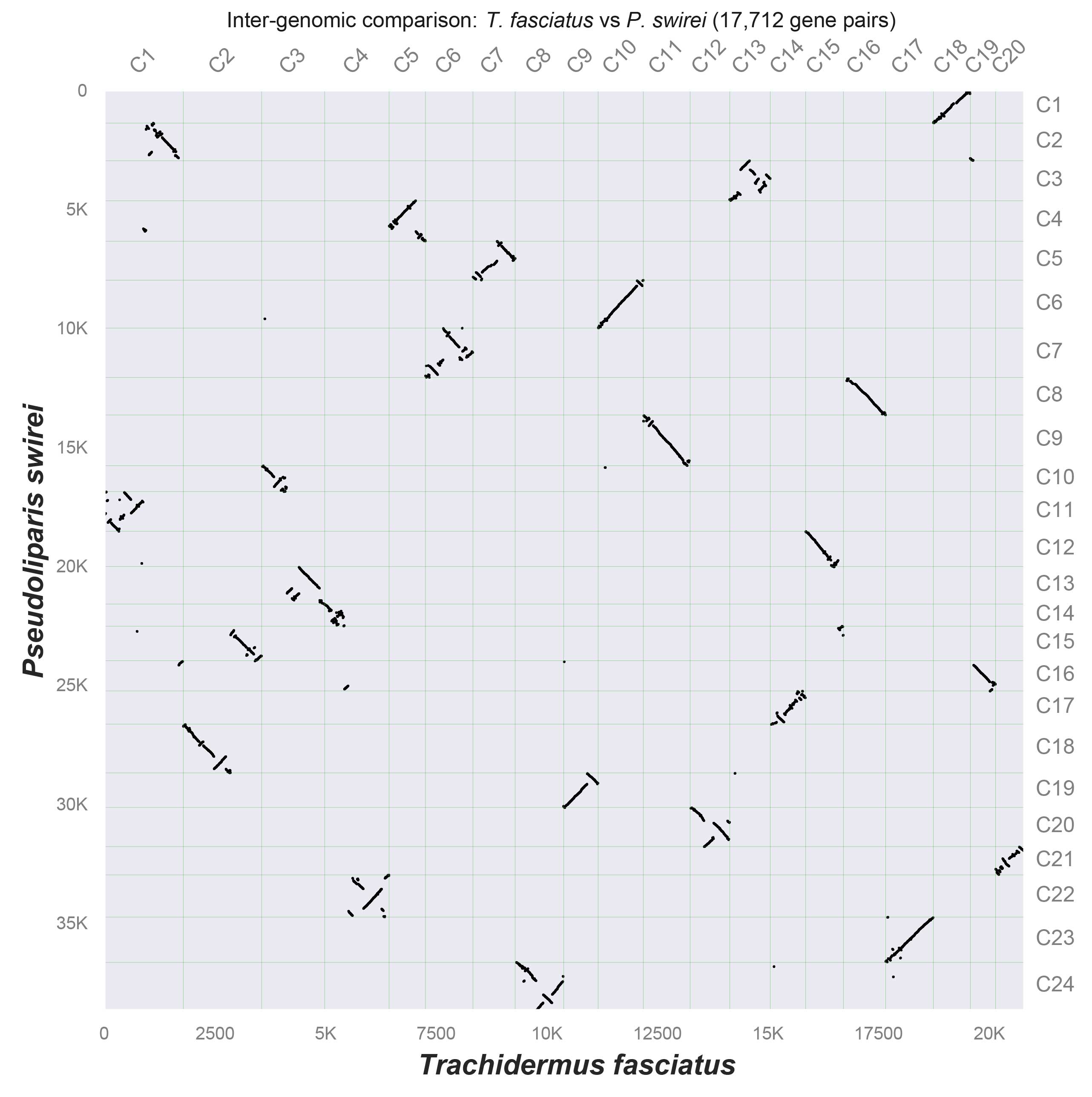


Figure. **S6.** Oxford grid comparison of roughskin sculpin and P. swirei, showing the synteny between chromosomes of the two species.


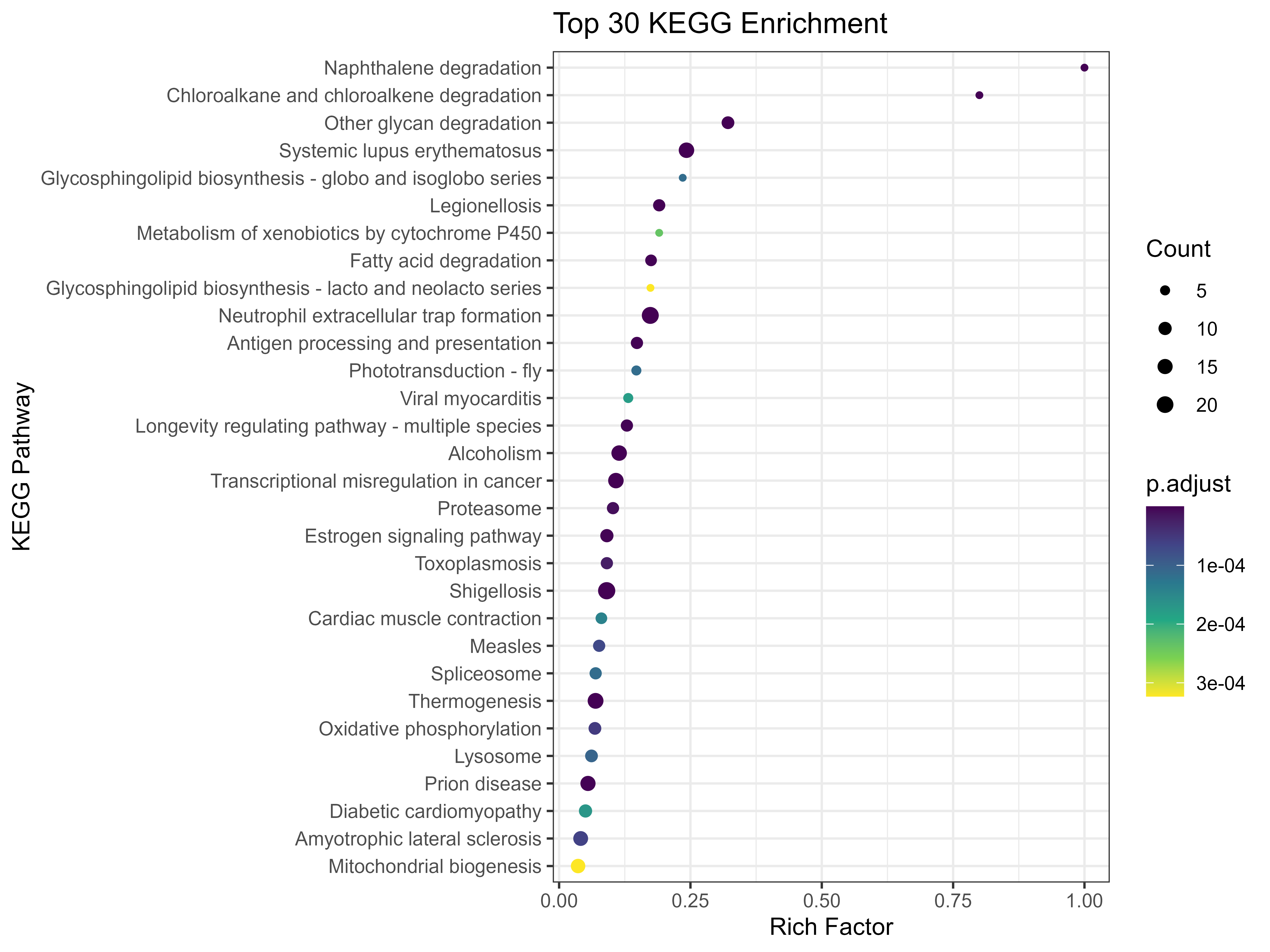


Figure. S7. Top 30 KEGG pathway enrichment results for genes in the expanded gene family of roughskin sculpin, with the horizontal axis representing the enrichment factor and the vertical axis representing the enriched KEGG pathways.


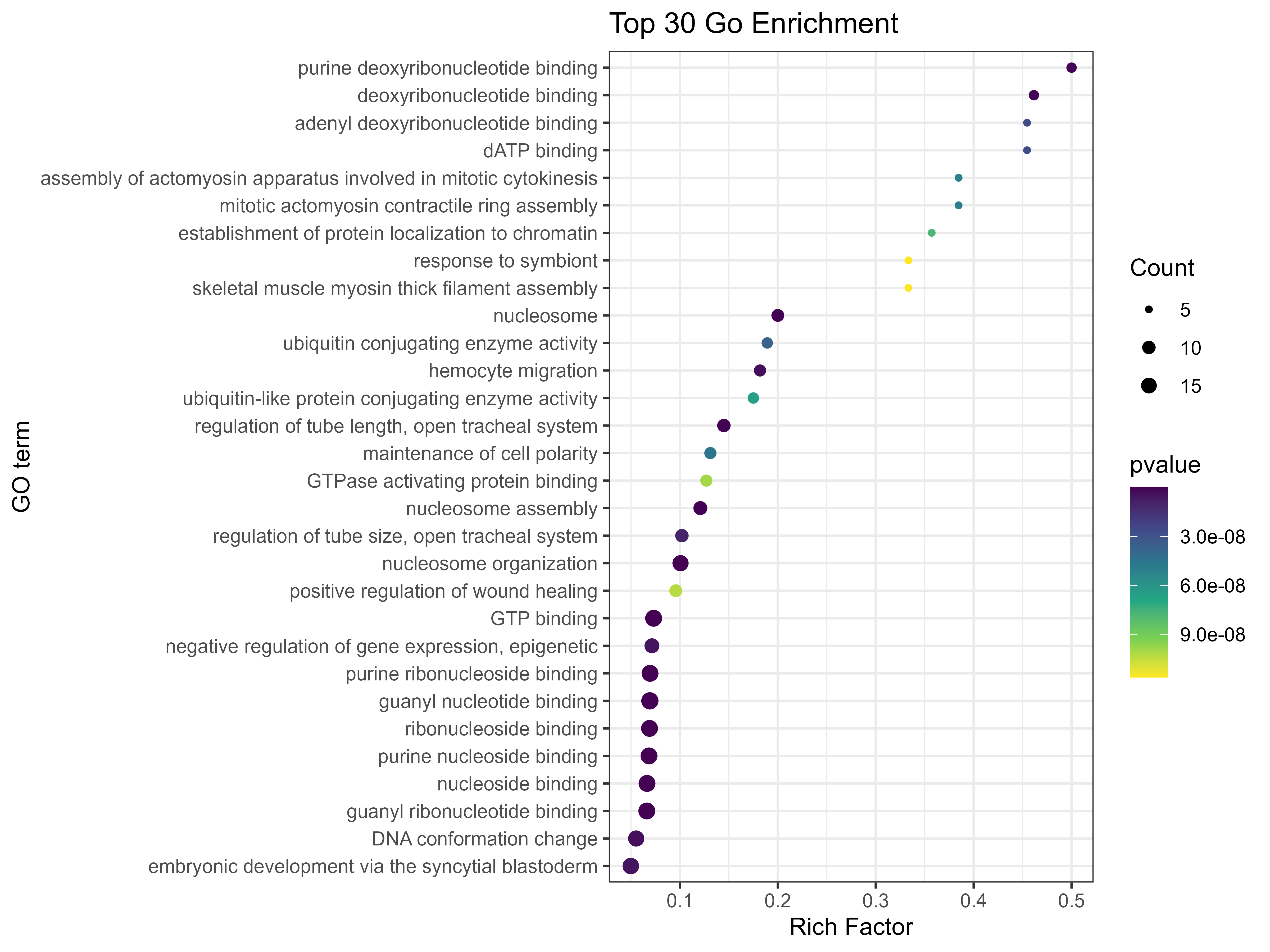


Figure. S8. Top 30 GO enrichment results of genes in the contracted gene family of roughskin sculpin, with the horizontal axis representing the enrichment factor and the vertical axis representing the enriched GO terms.


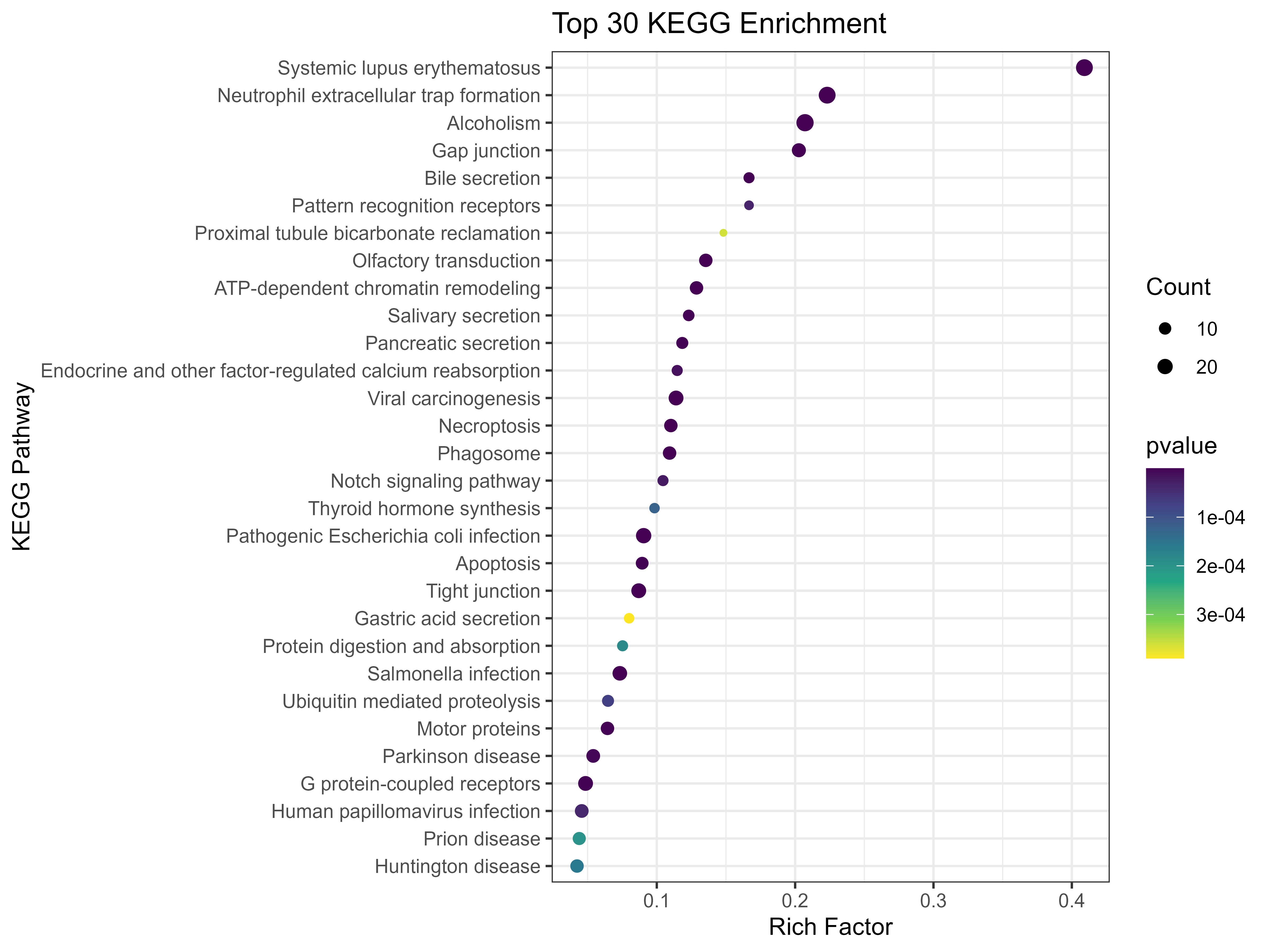


Figure. **S9.** Top 30 KEGG pathway enrichment results for genes in the contracted gene family of roughskin sculpin, with the horizontal axis representing the enrichment factor and the vertical axis representing the enriched KEGG pathwa
